# Fabrication of functionalized electrospun carbon nanofibers for enhancing lead-ion adsorption from aqueous solutions

**DOI:** 10.1038/s41598-019-55679-6

**Published:** 2019-12-19

**Authors:** Badr M. Thamer, Ali Aldalbahi, Meera Moydeen A, Abdullah M. Al-Enizi, Hany El-Hamshary, Mohamed H. El-Newehy

**Affiliations:** 1 Department of Chemistry, College of Science, King Saud University, Riyadh, 11451 Saudi Arabia; 2 Department of Chemistry, Faculty of Science, Tanta University, Tanta, 31527 Egypt

**Keywords:** Environmental sciences, Chemistry, Materials science, Nanoscience and technology

## Abstract

Electrospinning technique is a simple and cheap method for fabrication of electrospun nanofibers (ENFs), which in turn can converted into electrospun carbon nanofibers (ECNFs) by carbonization process. The controlling of the ECNFs properties (e.g. surface area, porosity, diameters) during fabrication, make it superior over the other carbon nanomaterials. The aim of our study is to modify the surface of ECNFs to increase its hydrophilicity and in turn its efficiency in removing lead ions (Pb^2+^) from aqueous systems. The surface modification was carried out in two steps starting from oxidation of pristine ECNFs to produce oxidized ECNFs (o-ECNFs), followed by covalently bonded of melamine, and poly(*m*-phenylene diamine) for forming melamine-functionalized ECNFs (melam-ECNFs) and poly(*m*-phenylene diamine)-functionalized ECNFs (P*m*PDA-ECNFs), respectively. The as-prepared materials were characterized in routine way. The ability of the as-prepared materials towards adsorption of Pb^2+^ ions as heavy metal was investigated with the study of some factors such as pH solution, contact time, initial concentration and temperature. The adsorption process was analyzed isothermally, and kinetically. According to the values of the thermodynamic parameters, the adsorption of Pb^2+^ ions onto the functionalized ECNFs was endothermic and spontaneous, except with melam-ECNFs was exothermic.

## Introduction

Environmental pollution with heavy metals is a major challenge at present and for the future. The continuation of this challenge lies in the dependence of many industries on these metals, as well as their accumulation in nature and their non-degradability^1^. Among toxic metals, the lead ion (Pb^+2^) is one of the most hazardous metals in the category of priority pollutants. The transfer of lead ions to organisms through the food chain can cause serious health problems (e.g., mental retardation, anemia, and kidney disease) owing to their accumulation and the inability of the body to eliminate them^2^. The maximum concentration of lead ions (Pb^2+^) in public drinking water is 15 μg/L, according to a report of the US Environmental Protection Agency^3^. Thus, lead ions should be removed from wastewater to avoid prospective hazards to environment and humans. Many techniques have been used to remove lead ions and other metal ions from aqueous solutions and wastewater, including filtration, precipitation, ion exchange, and adsorption^4^. Adsorption is one of the most favorable and economical techniques for removing toxic metals and other chemical contaminants from wastewater^5^. Therefore, the synthesis of new, effective, and economical adsorbent materials has been the goal of many researchers in the field of water treatment^6^.

Fibrous nanomaterials (FNMs) have received considerable attention in industrial and academic fields owing to their unique properties and possibility for large-scale production^7,8^. The large surface area, low density, highly porous structure, and distinctive mechanical properties of FNMs make them promising materials for many applications, such as filtration, scaffolds in tissue engineering, drug delivery, energy conversion, and environmental applications^9–11^. Recently, FNMs have been widely applied as nanoadsorbents for the removal of various pollutants from aqueous systems owing to their distinctive properties. Generally, FNMs are categorized into several types according to their composition: polymeric nanofibers, carbon nanofibers, inorganic nanofibers, and composite nanofibers. There are many techniques of producing FNMs, but the electrospinning is the most common technique for producing electrospun carbon nanofibers (ECNFs) because it is easy, simple, inexpensive and allows the large-scale production of fibers with multiple morphological^12–14^. ECNFs have been attracted considerable attention as efficient adsorbents for the removal of pollutants owing to their high surface-to-volume ratio and their ability to establish π-π electrostatic interactions. ECNFs have exhibited good adsorption capabilities for various nonpolar pollutants, such as toluene, benzene, and mineral oils^15–17^. However, pristine ECNFs as adsorbents is ineffective for the removal of ionic pollutants from aqueous systems because of their hydrophobic nature and the strong intermolecular interactions (Van der Waals forces) between the fibers, which could reduce the dispersion and lead to the formation of aggregates. A common technique for increasing the adsorption capacity of carbon nanomaterials is surface modification, which generates functional groups on their surface and enhances the hydrophilicity^18^. Previous studies indicated that the surface of ECNFs can be chemically modified to improve porosity and produce various functional groups that in turn improves their ability to remove ionic contaminants^19–21^. Many oxygen functional groups, hydroxyl groups (-OH), and carboxylic groups (-COOH) can be created simultaneously onto the fiber surface via chemical activation^19,22,23^. Owing to the reactivity of carboxylic groups with many other functional materials (e.g. diamines and polymers), they play as active points for further organic functionalization, as well as enhancing dispersion property and adsorption capacities of carbon nanomaterials^24–26^. Most of the previous studies were limited to modifying the surface of ECNFs using acidic or alkali agents, and did not yet focus on their organic functionalization^27^. Although these studies have indicated the effectiveness of activated ECNFs for the removal of ionic pollutants such as dyes^28,29^, but have not been utilized to remove toxic metals. The surface modification of ECNFs has the potential for improving their properties, and making those promising candidates for various applications, including heavy metals removal. Amino modification is a widely used method to produce nitrogen containing functional groups (e.g. –C-N, -C=N, -NH, -NH_2_) onto the surface of carbon nanomaterials, which can enhance their adsorption efficiency towards heavy metal ions removal^30^. Melamine and polyphenylene diamine are one of the amino reagents containing rich amino groups that can covalently bind to the surface of carbon materials and enhancing their efficiency in adsorption of lead ions^31,32^.

Herein, we report the first study on the preparation of amine-functionalized ECNFs and their use for removing Pb^2+^ ions from aqueous solutions. The prepared materials were achieved via electrospinning, thermal treatment, and acid treatment, followed by an amine organic functionalization process. The utilization of pristine, oxidized, and functionalized ECNFs for the removal of Pb^2+^ ions from an aqueous solution and the adsorption mechanism were investigated *via* isotherm, kinetic, and thermodynamic analyses.

## Results and Discussion

### Characterization of adsorbents

**Figure 1:**
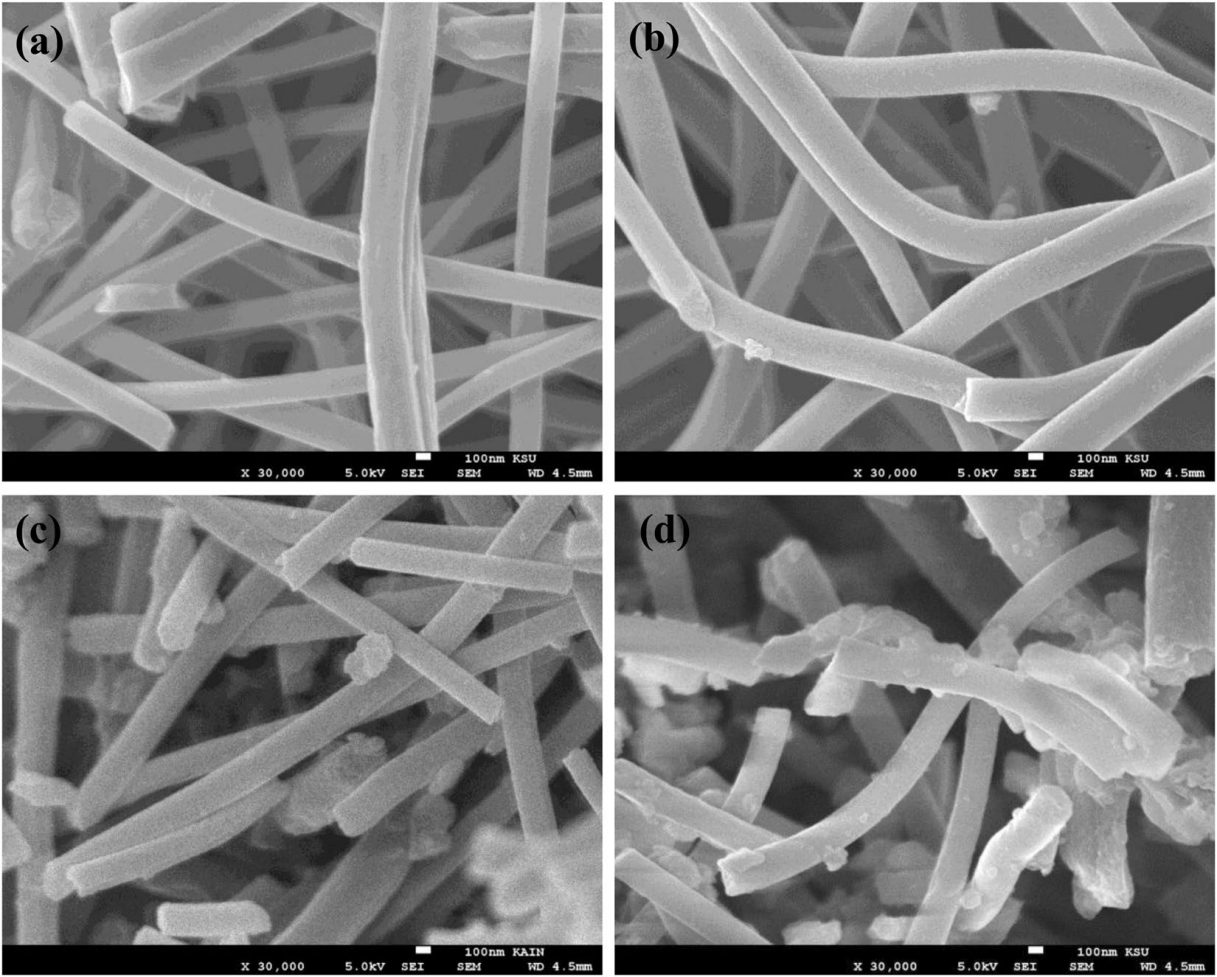
SEM image of ECNFs (**a**), o-ECNFs (**b**), melam-ECNFs (**c**), and P*m*PDA-ECNFs (**d**).

FESEM images of pristine and functionalized ECNFs are shown in Fig. 1. The pristine ECNFs kept its morphology with a smooth surface, and average fiber diameter of 229.3 nm (Fig. 1a). Upon oxidation, the surface of the o-ECNFs became rougher, as the acid treatment caused structural defects and created functional groups, which were expected to increase the amount of active sites and promote adsorption (Fig. 1b). The roughness increased with melam-ECNFs and the fiber diameter became larger (252 nm) due to the surface functionalization with melamine (Fig. 1c). After functionalization with P*m*PDA, nanoparticles were observed onto the surface of P*m*PDA-ECNF nanocomposites, confirming the successfully grafting of P*m*PDA onto the surface of the ECNFs (Fig. 1d). In general, compared with the pristine ECNFs, the structure of the functionalized ECNFs was partially broken, but their nanomorphologies were preserved.

**Figure 2:**
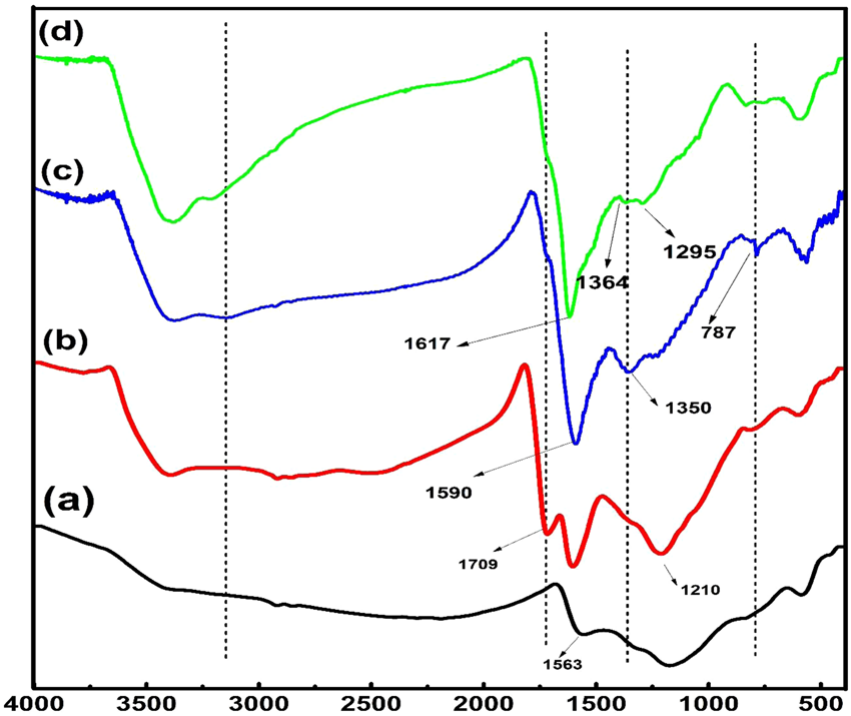
FTIR spectrum of ECNFs (**a**), o-ECNFs (**b**), melam-ECNFs (**c**), and P*m*PDA-ECNFs (**d**).

The new functional groups appeared on the surface of functionalized ECNFs were affirmed by FT-IR spectroscopy, as displayed in Fig. 2. The FT-IR spectrum of pristine ECNFs exhibited characteristic absorption peaks at 1563 cm^−1^ (C = C stretching from aromatic system) and 1174 cm^−1^ (C-N stretching vibration). The successful functionalization of the oxygenated functional groups onto the o-ECNFs surface was also confirmed by the new peaks at 3395 and 1709 cm^−1^, which were attributed to hydroxyl groups and carbonyl-carboxylic groups, respectively. The spectrum of the melam-ECNFs exhibited the disappearance of the absorption band of the carbonyl-carboxylic groups at 1709 cm^−1^ and the appearance of a new sharp peak at 1590 cm^−1^, which was ascribed to the formation of carbonyl-amide groups. Additionally, two new peaks observed at 1350 and 787.5 cm^–1^ due to the stretching frequencies and the out-of-plane bending modes of the triazine ring, respectively^33^. This confirms the grafting of melamine molecules onto the surface of the o-ECNFs *via* the creation of amide bonds. For P*m*PDA-ECNFs, new peaks appeared at 1617, 1364 and 1295 cm^−1^, which may be assigned to the stretching vibration of quinoid rings and benzenoid and quinoid imine units in poly(*m*-phenylene diamine), respectively^34^. Moreover, the absorption peak of the carbonyl-carboxylic group at 1709 cm^−1^ was shifted to 1617 cm^−1^ owing to its conversion into carbonyl-amide group. The absorption peak of the amide group overlapped with that of the quinoid rings. Thus, it can be inferred that P*m*PDA was successfully grafted onto the ECNFs.

**Figure 3:**
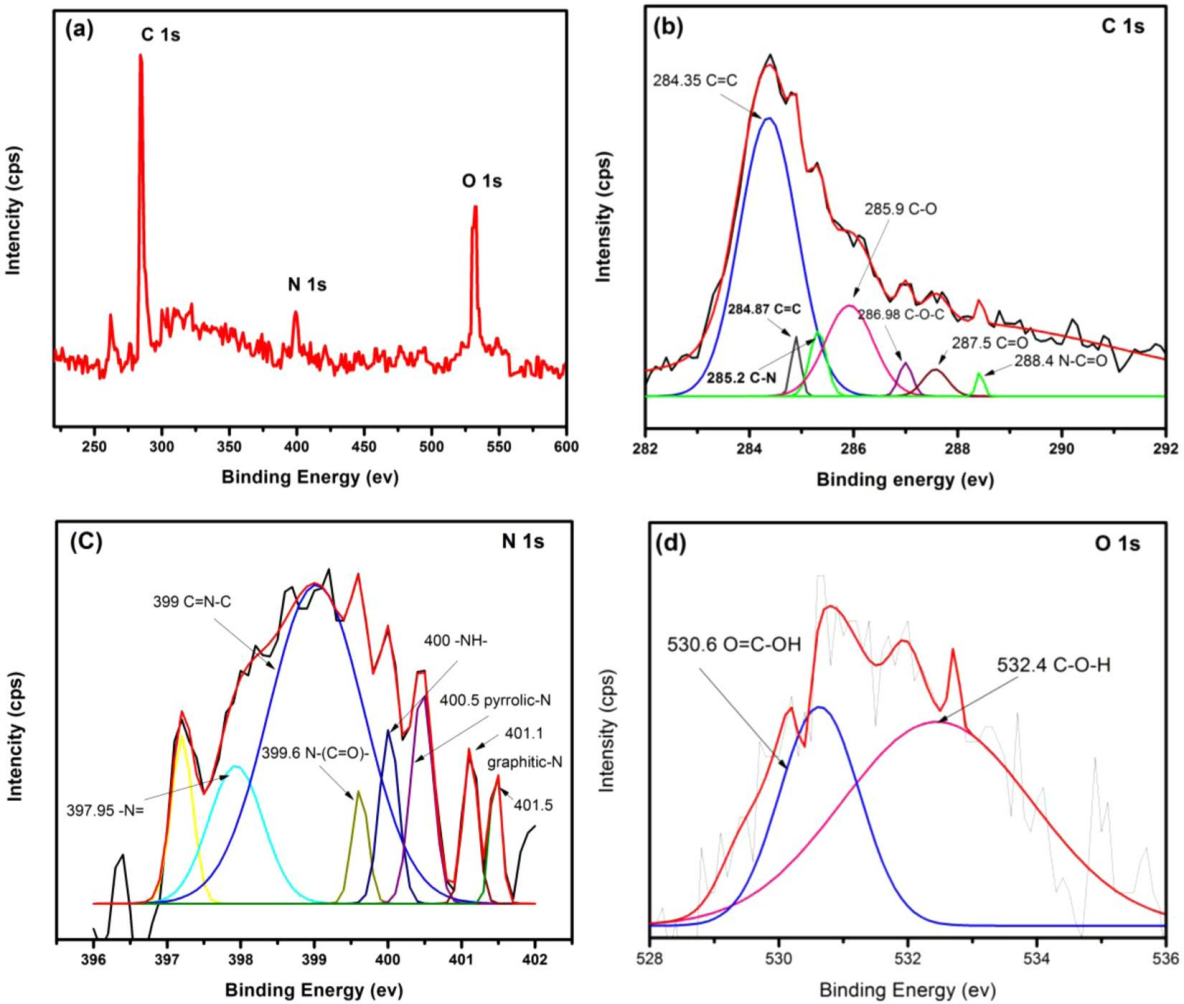
XPS spectra of melam-ECNFs.

**Figure 4:**
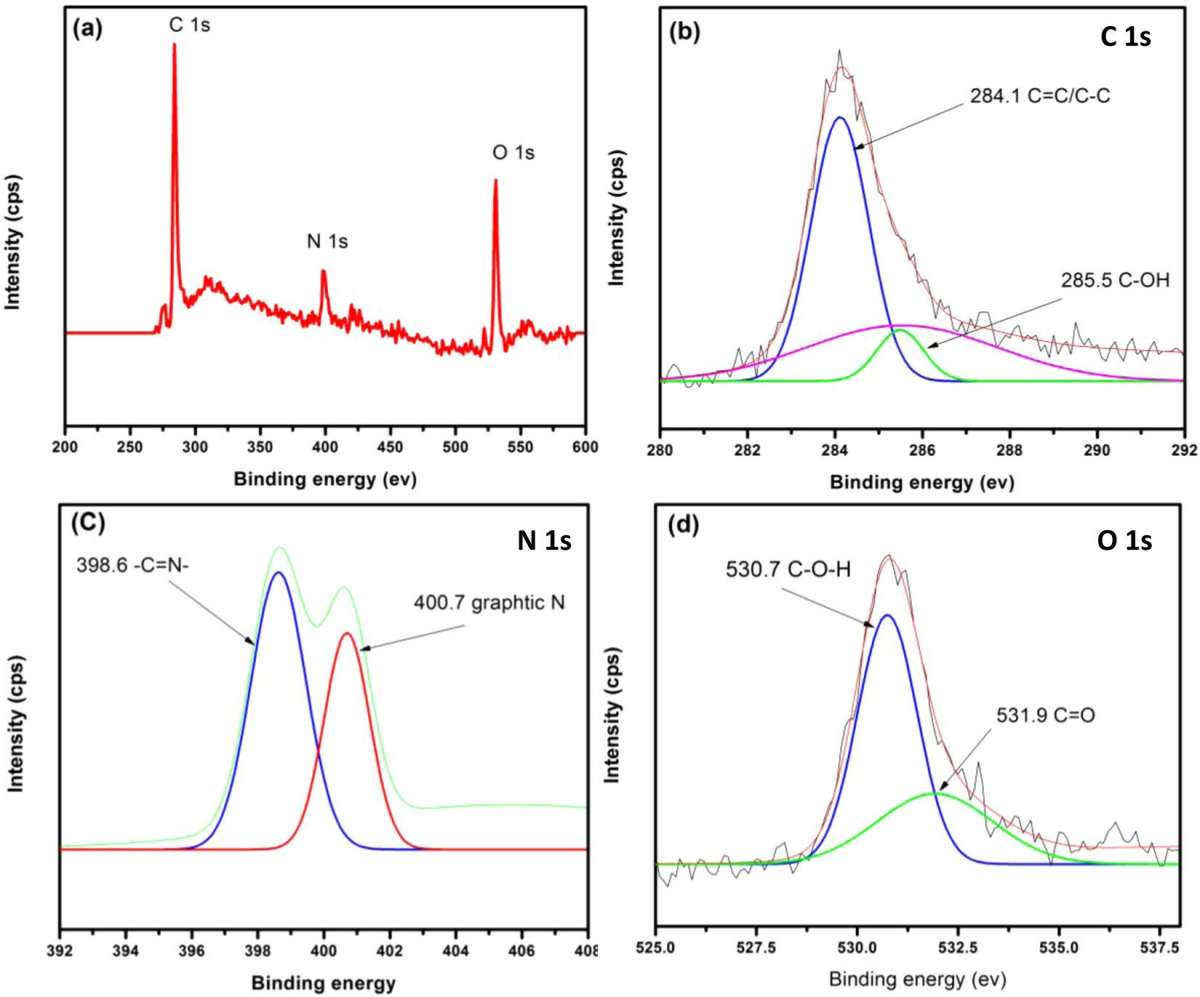
XPS spectra of P*m*PDA-ECNFs.

XPS spectra (wide range scan) of melam-ECNFs and P*m*PDA-ECNFs and high resolution spectra with the deconvolution for their relevant C1s, N1s and O1s are shown in Figs. 3 and 4. As displayed in Figs. 3a and 4a, the wide range scan reveals three distinct and sharp peaks at C 1s/~284.5 eV, N 1 s/~399 eV and O 1 s/~532 eV which indicated that the melam-ECNFs and P*m*PDA-ECNFs contained three elements: carbon, nitrogen and oxygen. The appearance of a nitrogen peak in both spectra is a physical evidence of successful functionalization process onto surface of ECNFs by melamine and P*m*PDA. To determine the nature of the functional groups associated with the surface of ECNFs after functionalization, deconvolutions of C 1 s, N 1 s and O 1 s were performed. The spectra of C 1 s for melam-ECNFs exhibits several peaks at 284.35, 284.87, 285.2, 285.9, 287.5 and 288.4 eV which attributed to C=C, C-C, C-N, C-O, C=O and N-C=O, respectively^35^. Although the peak of C=C (284.35 eV) is predominant, the appearance of other peaks confirms that the surface of mealm-ECNFs contains oxygen and nitrogen functional groups.

The N 1 s spectra shows the nitrogen takes many forms according to the nature of nitrogen functional groups that bonding with surface of ECNFs as shown in Fig. 3c. The main peak is located at 399 eV which is attributed to C=N-C that form melamine structure^36^. Two peaks in spectra of O 1 s with a binding energy of 530.6 and 532.4 eV in Fig. 3d are assigned to the O=C-OH and C-O-H, respectively. For P*m*PDA-ECNFs, the deconvolution analysis shows significant peaks at 284.1 (C=C) and 285.5 (C-OH) eV for C 1 s, 398.6 (C=N-) and 400.7 eV (graphitic N) for N 1 s and 530.7 (C-OH) and 531.9 eV (C=O) for O 1 s as shown in Fig. 4(b–d) ^37^. Based on the XPS analysis, the hydrophilicity of the functionalized ECNFs could be attributed to the presence of oxygen and nitrogen functional groups on their surface.

**Figure 5:**
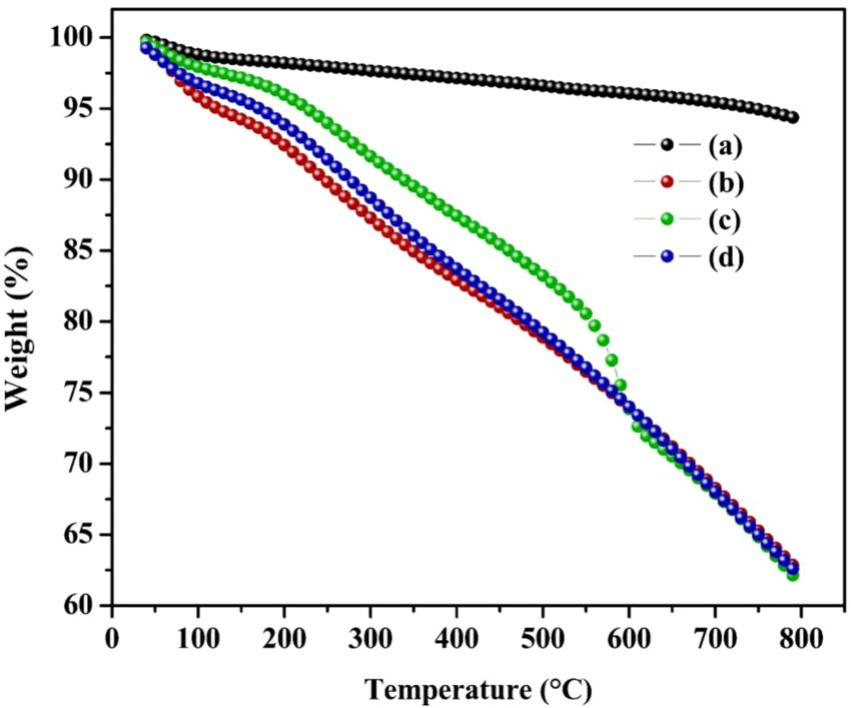
TGA thermogram of ECNFs (**a**), o-ECNFs (**b**), melam-ECNFs (**c**) and P*m*PDA-ECNFs (**d**).

For further investigation of the grafting process on the surface of the ECNFs, TGA was performed under N_2_ gas as shown in Fig. 5. The residual weight percentage of pristine ECNFs at 600 °C was 96%, indicating its high thermal stability. After surface modification, the thermal stability differed substantially, and the residual weight percentages of o-ECNFs, melam-ECNFs, and P*m*PDA-ECNFs at 600 °C were 74%, 78%, and 75%, respectively. As shown in Fig. 5, the weight loss of the o-ECNFs at 120 °C is approximately 5.0%, which may be ascribed to the evaporating moisture. The major steps of the weight loss of o-ECNFs occurred within the temperature ranges of 130–410 and 420–600 °C which may be assigned to the removing of oxygen functional groups^27^. The melam-ECNFs showed different thermal decomposition behavior and high stability compared to o-ECNFs. Thermal degradation process of melam-ECNFs was occurred in three steps. The first step started below 120 °C due to the evaporation of physically adsorbed water. The second step was occurred at 210–360 °C which attributed to the degradation of oxygen functional groups (OH and COOH). The final step was occurred over 500 °C which may be assigned to the decomposition of melamine and intermediate compounds. The P*m*PDA-ECNFs showed more stability than o-ECNFs and was degraded in three steps. The first degradation observed at 30–130 °C was mainly due to the evaporation of water molecules and the second degradation was observed at 130–230 °C was due to the deprotonation of polymeric chains which may lead to the losing of dopant HCl^34^. The third degradation observed beyond at 600 °C was attributed to the degradation of polymer chains. The significant differences in thermal behavior clearly confirmed the surface modification of ECNFs achieved by the oxidation and grafting process.

The surface area and pore volume of ECNFs, o-ECNFs and P*m*PDA-ECNFs are listed in Table S1. The surface area of P*m*PDA-ECNFs is evidently lower than the o-ECNFs. This may be due to the blocking of pores upon grafting with P*m*PDA, which in turn decreases the surface area of the ECNFs. On the other hand, the functionalization of ECNFs with melamine and P*m*PDA plays an important role in enhancement of the adsorption capacity which is not depend only from the surface area.

**Figure 6:**
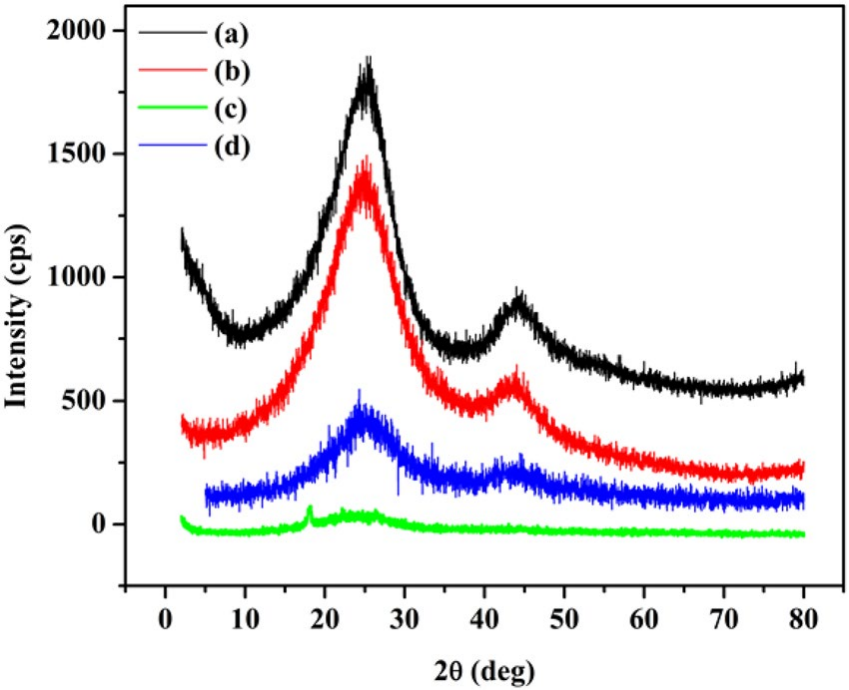
XRD patterns of ECNFs (**a**), o-ECNFs (**b**), melam-ECNFs (**c**) and P*m*PDA-ECNFs (**d**).

The study of the change in the crystalline structure of carbon materials *via* XRD is useful for confirming surface modification. As shown in Fig. 6, the XDR spectrum of the pristine ECNFs exhibited a main peak at 24.4° and a weak peak at 44.1°, which are related to the pseudo-graphite structure and the turbostratic carbon structure, respectively. After functionalization, the intensity of the main peak decreased, and the diffraction angle of the peak increased. The average interlayer spacing (*d*_002_) of the ECNFs was 0.364 nm, which was calculated using Bragg’s equation. After functionalization, the o-ECNFs, melam-ECNFs, and P*m*PDA-ECNFs exhibited gradually decreasing with brooding of peaks and reducing in the average *d*_002_ values, which were calculated as 0.361, 0.359, and 0.353 nm, respectively. The decreasing and widened graphitic peak of the functionalized ECNFs is attributed to a breakdown in the graphitic lattice structure. Moreover, the spectrum of the melam-ECNFs exhibited a new peak at 18.08°, which corresponds to the triazine unit^38^. In general, the weaker diffraction peaks for functionalized ECNFs are due to the lower-order structure of the carbon fiber graphite layers, which confirms the successful modification of the fiber surface.

### Adsorption study

#### Effect of pH

**Figure 7:**
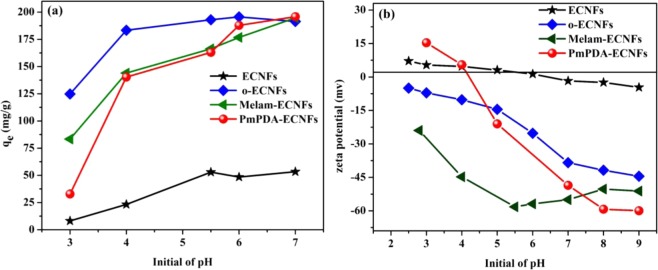
(**a**) pH effect on the adsorption of Pb^2+^ ions onto prepared adsorbents; (**b**) pH effect on Zeta potential values.

The pH has a significant influence on the adsorption process because of its effects on both the adsorbent surface charge and the solubility of metal ions. For lead ions, it can be found in four forms according to the pH of the aqueous solution: as Pb^2+^ at pH < 6.0, and at a higher pH as Pb(OH)^+^, Pb(OH)_2_, and Pb(OH)^3−^^39^. The lead is permanently present in the Pb^2+^ ions form at a pH of <6.0, while the other forms are produced at a higher pH. At a pH of >7, the precipitation of lead occurred; thus, the adsorption of lead ions is favored occurred in acidic media^40^. Figure 7a shows the influence of the pH on the adsorption of Pb^2+^ ions onto the surface of the introduced adsorbents. The results shown that Pb^2+^ ions adsorption onto functionalized ECNFs was highly dependent on the pH owing to its effect on the ionization of the functional groups linked with the fiber surface. It was found that the functionalized ECNFs exhibited better adsorption capacity with increasing the pH value, while the pristine ECNFs were only slightly pH-dependent. The optimum pH value was determined as 5.5 to ensure that Pb^2+^ ions are not precipitated in solution and this value was used throughout all the adsorption experiments.

To study the charge nature on the pristine and functionalized ECNFs surfaces, the zeta potential was measured at different pH values (3 to 9), as shown in Fig. 7b. The surfaces of the o-ECNFs and melam-ECNFs were negatively charged in the tested pH range because of the effects of oxygen and amino functional groups. The P*m*PDA-ECNFs exhibited different behavior: their surface was positively charged at pH < 4.2 and became negatively charged at higher pH values. At a low pH, the amount of Pb^2+^ ions adsorbed onto the o-ECNFs and melam-ECNFs was small, despite the negatively charged surfaces, owing to the increasing in the concentration of H_3_O^+^ ions, which competed with Pb^2+^ ions at the adsorption sites. In contrast, the adsorption of Pb^2+^ ions increased at high pH was due to the increase of negative charge onto the adsorbent surface and the decrease of H_3_O^+^ concentration compared to Pb^2+^ ions. Hence, the adsorption of Pb^2+^ ions onto functionalized ECNFs was promoted by the electrostatic interaction between the oxygen and amino groups on their surface and Pb^2+^ ions at high pH. The difference in the adsorption capacity can be explained by changing the pH through the following reactions.

1 $$-{\rm{COOH}}+{{\rm{H}}}_{3}{{\rm{O}}}^{+}\to -\,{{{\rm{COOH}}}_{2}}^{+}+{{\rm{H}}}_{2}{\rm{O}}$$

2 $$-{\rm{NH}}-+{{\rm{H}}}_{3}{{\rm{O}}}^{+}\to {-}^{+}{{\rm{NH}}}_{2}-+{{\rm{H}}}_{2}{\rm{O}}$$

3 $$-{\rm{COOH}}+{\rm{OH}}\to -{{\rm{COO}}}^{-}+{{\rm{H}}}_{2}{\rm{O}}$$

4 $$-{\rm{N}}{\rm{H}}-+{\rm{O}}{\rm{H}}\to -\,{{\rm{N}}}^{{\textstyle \text{\_-}}}+{{\rm{H}}}_{2}{\rm{O}}$$

According to the small quantities of the functionalized ECNFs and their high adsorption capacity at pH values of 5–6, they can be used as effective and suitable adsorbents to remove lead ions from contaminated water.

### Isotherm study

**Figure 8:**
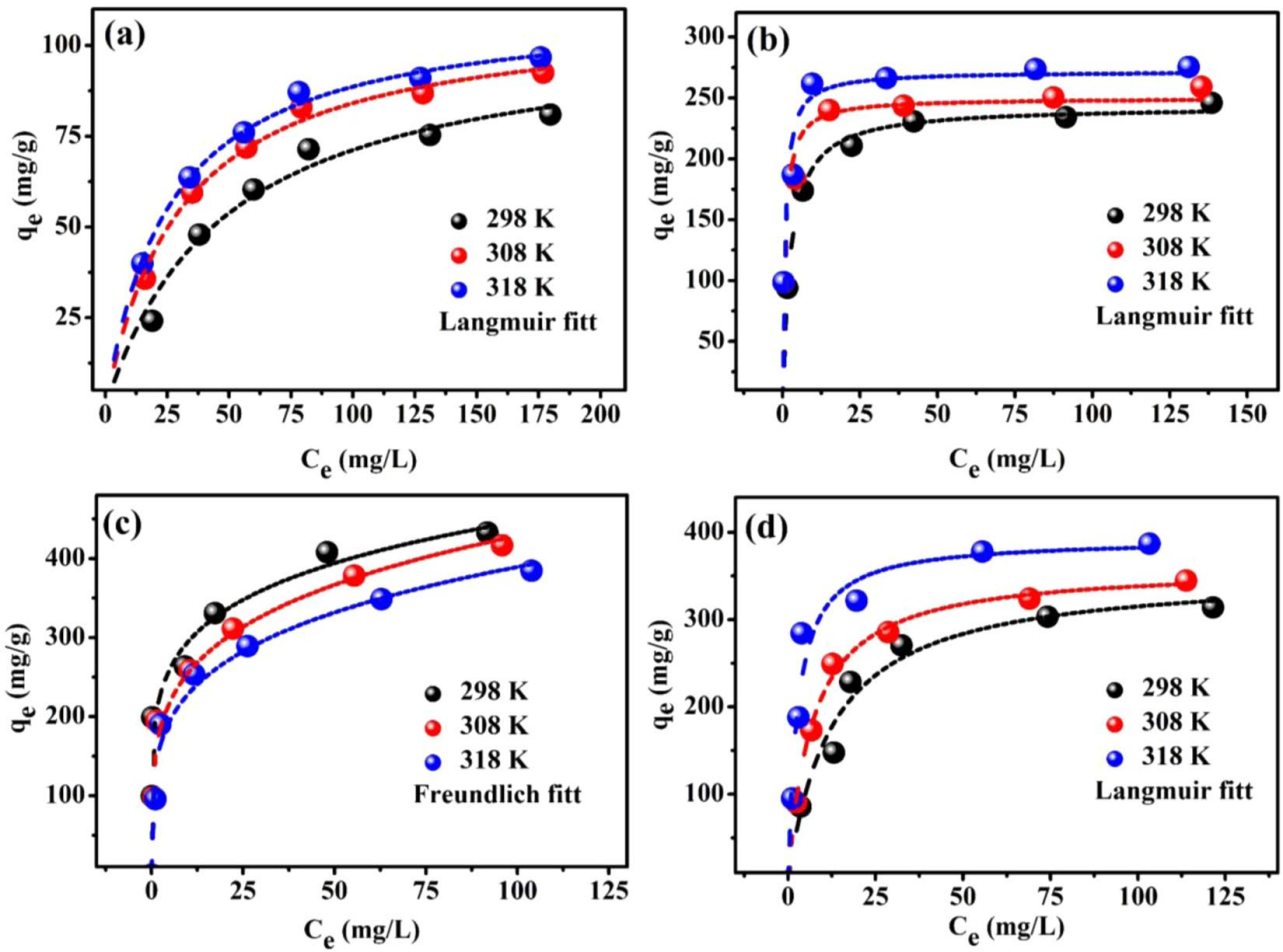
Isotherms for the adsorption of Pb^2+^ ions onto ECNFs (**a**), o-ECNFs (**b**), melam-ECNFs (**c**) and P*m*PDA-ECNFs (**d**).

Three adsorption models were employed to investigate the adsorption behavior of the pristine and functionalized ECNFs: Langmuir^41^, Freundlich^42^, and Dubinin–Radushkevich (D–R)^43^. Equations and parameters of all models are shown in Supporting Information (Section I). The nonlinear optimization method was used to reduce the respective error, and the *R*^2^ and *χ*^2^ values were used to identify the best-fit model for the adsorption process. Figures 8 and S1 show the equilibrium isotherms of Pb^2+^ ions onto pristine and functionalized ECNFs at various temperatures (298, 308, and 318 K). Clearly, the amount of Pb^2+^ ions adsorbed onto the functionalized ECNFs at equilibrium was significantly higher than that for the pristine ECNFs. This was attributed to functional groups linked with surface of the functionalized ECNFs, which provided sufficient active sites for adsorbing lead ions, reduced the diffusion resistance, and improved dispersibility of functionalized ECNFs. Additionally, the maximum adsorption capacity of Pb^2+^ ions on all adsorbents was increased with increasing the temperature, except for melam-ECNFs, which favored the adsorption at low temperature. The exothermic nature of adsorption onto surface of melam-ECNFs may be attributed to the presence of a large number of adsorption sites, which is consistent with previous research^44^.

**Figure 9:**
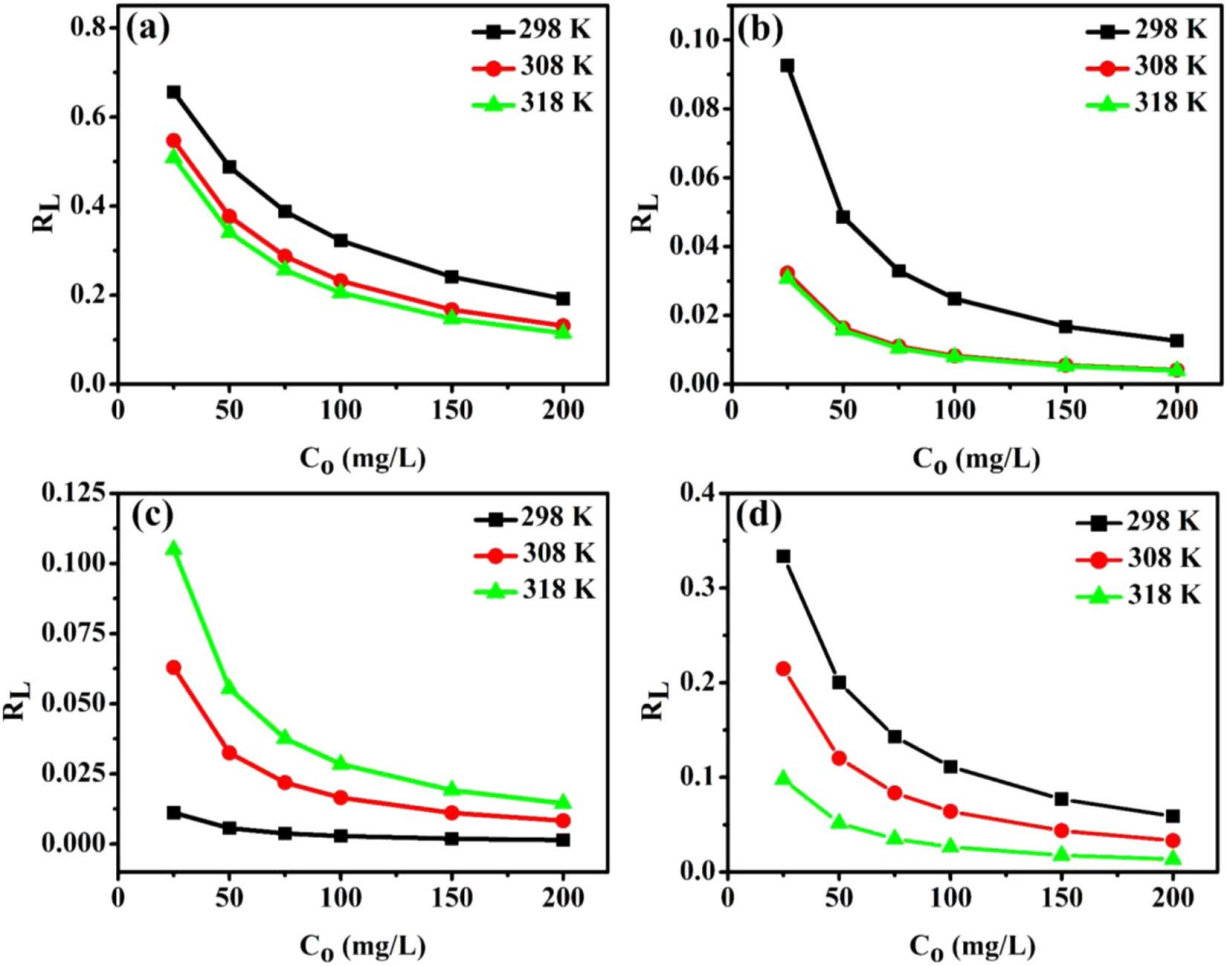
Separation factor (*R*_*L*_) with respect to the initial concentration of Pb^2+^ ions on ECNFs (**a**), o-ECNFs (**b**), melam-ECNFs (**c**) and P*m*PDA-ECNFs (**d**).

Table 1 presents the parameters of the applied nonlinear isotherm models for analyzing the adsorption process at different temperatures. The maximum monolayer adsorption capacity (mg/g) of Pb^2+^ ions onto the introduced adsorbents at 298 K decreased in the following order: melam-ECNFs (367.5) > P*m*PDA-ECNFs (354.5) > o-ECNFs (243) > ECNFs (105). As indicated by the high values of *R*^2^ and low values of *χ*^2^, the Langmuir model fitted the experimental data, which was better than the Freundlich and D–R models, except for the experimental data for the melam-ECNFs, which were well fitted by the Freundlich model. This clearly indicates that the adsorption of Pb^2+^ ions onto the surface of melam-ECNFs was heterogeneous, whereas it was homogeneous for the other adsorbents. The separation factor (*R*_*L*_) is a useful parameter for predicting the favorability of the adsorption process and is expressed as follows^43^:

5 $${R}_{L}=\frac{1}{1+{K}_{L}{C}_{o}}$$

where *C*_*o*_ represents the initial concentration (mg/L), and *K*_*L*_ represents the Langmuir constant (L/mg). Adsorption is favorable when 0 < *R*_*L*_ < 1 and is irreversible when *R*_*L*_ = 0. The *R*_*L*_ values for the adsorption of Pb^2+^ ions onto all the adsorbents at different temperatures are calculated and plotted, as shown in Fig. 9. The *R*_*L*_ valuesranged between 0 and 1.0 for all adsorbents and decreased with increasing the initial concentration of Pb^2+^ ions. This indicates the favorability of adsorption process in the range of concentrations studied. Furthermore, with the increasing adsorption temperature, the *R*_*L*_ value gradually decreased, except for the melam-ECNFs (it increased) which indicates that the adsorption of Pb^2+^ ions onto the melam-ECNFs was highly favorable at a low adsorption temperature^45^. For the Freundlich model, the values of *n* were <1 for all adsorbents at different temperatures, suggesting that the adsorption was favorable. For D–R model, the calculated *E* values for the all adsorbents were <8 kJ∙mol^−1^, confirming that the adsorption of Pb^2+^ ions onto the pristine and functionalized ECNFs was physical. The values of *R*_*L*_, *n*, and *E* indicated that the adsorption of Pb^2+^ ions onto all adsorbents was favorable.

**Table 1:** Adsorption isotherm parameters for Pb^2+^ ions with pristine and functionalized ECNFs as adsorbents.

	Langmuir				Freundlich				D–R				
	*Q*°_*max*_	*K*_*L*_	*R*^2^	*χ*^2^	*K*_*F*_	n	*R*^2^	*χ*^2^	*Q*_*DR*_	*K*_*DR*_	*R*^2^	*χ*^2^	*E*
**298 K**													
ECNFs	105.10	0.0210	0.9843	1.65	10.57	0.40	0.9454	5.24	76.49	87.30	0.9712	3.17	0.0756
o-ECNFs	243.11	0.3919	0.9903	0.6166	116.39	0.16	0.8425	13.12	223.75	0.5998	0.8480	9.53	0.9130
melam-ECNFs	367.54	3.55	0.8666	51.04	203.76	0.17	0.9650	28.84	361.03	0.0418	0.8593	51.74	3.46
P*m*PDA-ECNFs	354.52	0.0799	0.9734	9.96	82.89	0.29	0.9201	30.33	312.85	20.82	0.8910	1541.60	0.1549
**308 K**													
ECNFs	109.51	0.0331	0.9957	0.3640	17.98	0.32	0.9654	3.22	85.57	43.47	0.9599	3.48	0.1072
o-ECNFs	249.75	1.20	0.9376	5.95	144.29	0.13	0.8300	14.98	237.29	0.0950	0.9391	12.73	2.29
melam-ECNFs	371.63	0.5961	0.9254	20.82	161.67	0.21	0.9769	17.44	341.22	0.2350	0.8696	37.15	1.45
P*m*PDA-ECNFs	361.19	0.1463	0.9960	1.43	110.14	0.25	0.9387	27.80	303.14	2.5200	0.9049	80.08	0.4454
**318 K**													
ECNFs	111.49	0.0387	0.9974	0.1952	21.15	0.30	0.9707	2.72	89.02	32.46	0.9571	3.61	0.1241
o-ECNFs	271.88	1.26	0.9211	10.59	159.32	0.13	0.7676	25.00	255.66	0.0722	0.7785	18.26	2.63
melam-ECNFs	362.23	0.3412	0.9724	10.47	140.89	0.22	0.9719	17.50	319.04	0.4902	0.9100	33.87	1.01
P*m*PDA-ECNFs	392.52	0.3693	0.9597	21.12	161.54	0.20	0.8874	61.09	358.66	0.7404	0.9466	39.95	0.8217

### Kinetic study

**Figure 10:**
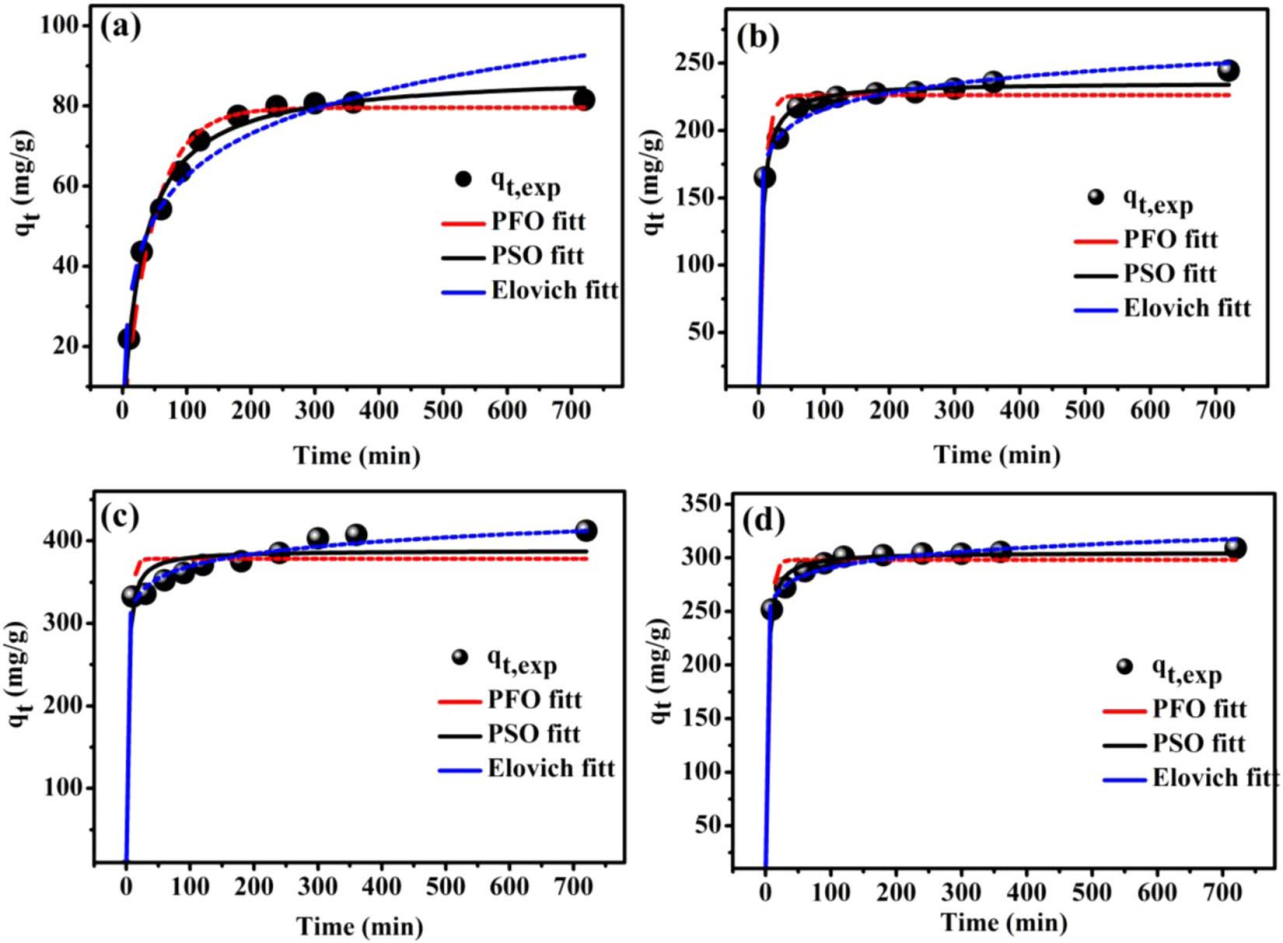
Nonlinear kinetic study of the adsorption of Pb^2+^ ions onto ECNFs (**a**), o-ECNFs (**b**), melam-ECNFs (**c**) and P*m*PDA-ECNFs (**d**).

Adsorbents with a high adsorption rate are valuable because they reduce the time required to complete the adsorption process. Therefore, studying the influence of time on the adsorption process is practically important for designing a treatment plant for contaminated water. Figures 10 and S2 show the contact time versus the adsorption capacity of the pristine and functionalized ECNFs. Clearly, the adsorption process was significantly affected by the functionalization process. This phenomenon was due to the presence of functional groups on the surface of the fibers, which enhanced the hydrophilicity of the fiber surface. Therefore, 3.0 and 2.0 h were required to reach the equilibrium for the adsorption of Pb^2+^ ions onto the pristine and functionalized ECNFs, respectively. To investigate the adsorption kinetics, three nonlinear models pseudo-first-order (PFO)^46^, pseudo-second-order (PSO)^47^, and Elovich^48^ were applied. All equations of models and parameters are shown in Supporting Information (Section II). Fig. 10 shows the fitting of the PFO, PSO, and Elovich models, and Table 2 presents the kinetic parameters and the *R*^2^ and *χ*^2^ values obtained *via* the nonlinear fitting. The *R*^2^ and *χ*^2^ values of the PSO model were higher and lower, respectively, than those of the other models. Moreover, the calculated adsorption capacity values (*q*_*e,cal*_) of the PSO model were closer to the experimental results (*q*_*e,exp*_) than those of the PFO model. However, according to the *R*^2^ (0.995) and *χ*^2^ (1.54) values, the Elovich model was more appropriate than the other models for describing the adsorption of Pb^2+^ ions onto the surface of the melam-ECNFs. This indicates that the surface of the melam-ECNFs was heterogeneous and that chemical adsorption may have occurred. In a heterogeneous system, chemisorption, ion exchange, and intraparticle diffusion can occur simultaneously, making it difficult to identify the dominant mechanism. For the Elovich model, *α* value of the functionalized ECNFs was higher than that of pristine ECNFs, confirming that the adsorption rate was enhanced after surface modification of the ECNFs.

**Table 2:** Parameters of nonlinear kinetic models for the adsorption of Pb^2+^ ions onto the pristine and functionalized ECNFs at 298 K.

	ECNFs	o-ECNFs	melam-ECNFs	P*m*PDA-ECNFs
**PFO**				
*q*_*e,exp*_	81.52	244.28	412.20	309.11
*q*_*e, cal*_	79.57	226.28	378.35	298.19
*k*_1_	0.0217	0.1195	0.2065	0.1815
*R*^2^	0.9822	0.9705	0.9550	0.9865
*χ*^2^	4.18	5.85	14.36	3.31
**PSO**				
*q*_*t, cal*_	88.28	235.55	388.49	305.21
*K*_2_ (×10^−4^)	3.55	8.83	10.90	0.0014
*R*^2^	0.9943	0.9941	0.9731	0.9971
*χ*^2^	0.5855	1.21	8.77	0.7436
**Elovich**				
*α*	3.66	40372.04	5.66*E6	2.55*E8
*β*	0.0075	0.05727	0.0462	0.0735
*R*^2^	0.9583	0.99165	0.9954	0.9963
*χ*^2^	4.74	1.79000	1.54	0.9294
**Intraparticle diffusion**				
*K*_*id*(1)_	6.13	11.33	4.55	6.28
*I*	5.85	130.18	316.26	235.27
*R*^2^	0.9711	0.9921	0.9671	0.9633
*K*_*id*(2)_	0.2368	1.31	0.8774	0.4887
*I*	75.74	209.46	388.94	295.94
*R*^2^	0.4976	0.9734	0.7980	0.9585

**Figure 11:**
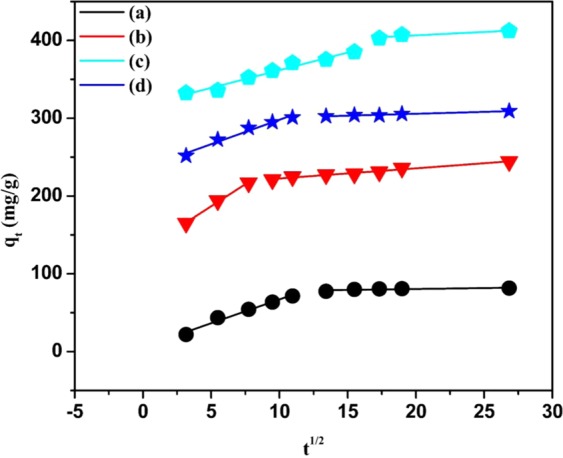
Intraparticle diffusion model for the adsorption of Pb^2+^ ions onto ECNFs (**a**), o-ECNFs (**b**), melam-ECNFs (**c**) and P*m*PDA-ECNFs (**d**).

To investigate the adsorption mechanism and identify the rate-determining step, an intraparticle diffusion model was applied, which is expressed as follows:

6 $${q}_{t}={K}_{id}{t}^{0.5}+C$$

where *k*_*id*_ (mg/g min^1/2^) and *C* (mg/g) represent the intraparticle diffusion rate constant and a constant related to the thickness of the boundary layer, respectively, and it can be determined according to the slope and intercept of the *q*_*t*_ vs. *t*^*0.5*^ plot. All factors of these models were calculated, as shown in Table 2. As shown in Fig. 11, the relationship between *q*_*t*_ and *t*^1/2^ is multilinear (two steps) during the adsorption, indicating that the adsorption process is complex and controlled by more than one mechanism. The first step represents the rapid adsorption of Pb^2+^ ions onto the active sites of the fiber surface, and the second step represents diffusion of Pb^2+^ ions from the surface to the internal pores. During the first step, most Pb^2+^ ions were rapidly adsorbed onto the exterior surfaces of the pristine and functionalized ECNFs, and the values of *K*_*id*(1)_ ranged between 4.55 and 11.33, indicating that the mass transfer occurred in the diffusion boundary layer^49^. In the second step, the adsorption was gradually, indicating that the intraparticle diffusion process was limited. Additionally, the intraparticle diffusion rate (*K*_*id*(2)_) was decreased sharply owing to the minimal pores and lower Pb^2+^ ions concentration in the solution. Moreover, the *Ci* value of the pristine ECNFs was lower than that of the functionalized ECNFs, suggesting that the boundary layer in the pristine ECNFs was thinner than that in the functionalized ECNFs. These results confirmed that the functionalization process enhanced the amount of active sites onto the surface of the ECNFs and improved its efficiency in removing Pb^2+^ ions.

### Proposed adsorption mechanism

The adsorption Pb^2+^ ions onto pristine ECNFs is non-specific binding, while on o-ECNFs, melam-ECNFs and P*m*PDA-ECNFs is a combined process. Based on the pH effect, the electrostatic interaction plays vital role in the adsorption process because of the amount of Pb^2+^ ions was increased by increasing value of pH. This electrostatic interaction may be attributed to the presence of functional groups such as –COOH, -NH_2_, =N- and –OH onto the surface of the functionalized ECNFs leading to increasing of Pb^2+^ ions adsorption.

The low adsorption capacity of pristine ECNFs is also crucial evidence of the role of these functional groups in the adsorption process. In addition, it can consider electrostatic interaction as the main factor here, as the surface charges of the functionalized ECNFs are negative as confirmed by zeta potential measurement. As shown in Fig. 11, the intraparticle diffusion of Pb^2+^ ions on functionalized ECNFs shows two straight lines without passing through the origin, which would suggest that the rate-limiting step is considered to be a combination of film diffusion and intraparticle mechanisms in the adsorption. In summary, the adsorption mechanism could be attributed to the functional groups on the surface of functionalized ECNFs, which could bind with Pb^2+^ ions through complexation or electrostatic interaction, while the adsorption mechanism on the surface of pristine ECNFs could be ascribed to nonspecific binding.

### Thermodynamic study

For further investigation for the nature of lead adsorption onto the ECNFs, o-ECNFs, ECNFs-melam, and P*m*PDA-ECNFs, the thermodynamic parameters, such as the standard enthalpy (∆*H*°), Gibbs free energy (∆*G*°), and standard entropy (∆*S*°) were studied and estimated. Thermodynamic Equations are shown in Supporting Information (Section III).

Table 3 presents the values of all thermodynamic parameters. As indicated by the negative values of ∆*G*°, the lead adsorption on the functionalized ECNFs was spontaneous and favorable. In contrast, the lead adsorption onto the pristine ECNFs was nonspontaneous, as indicated by the positive values of ∆*G*° at different temperatures. The positive values of ∆*H*° indicate that the adsorption of Pb^2+^ ions onto the ECNFs, o-ECNFs, and P*m*PDA-ECNFs was endothermic, whereas with the melam-ECNFs was exothermic (negative values of ∆*H*°). This is consistent with our experimental results presented in Table 1, where the adsorption capacity increased with the temperature for all adsorbents except the melam-ECNFs. The positive values of ∆*S*° indicate random increase at the solid–liquid interface during the adsorption of Pb^2+^ ions onto all the adsorbents, except for the ECNFs (random decrease). The negative value of ∆*S*° indicates that the randomness at the solid–liquid interface was reduced during the Pb^2+^ ions adsorption onto the melam-ECNFs and that the adsorption process occurred without structural changes in the melam-ECNFs^50^.

**Table 3:** Thermodynamic parameters for the adsorption of Pb^2+^ ions onto the pristine and functionalized ECNFs.

T (K)	van’t Hoff equation	*K*_*c*_	Δ*G*° (kJ/mol)	Δ*H*° (kJ/mol)	Δ*S*° (J/mol)
**ECNFs**					
298	y = −1870.93x + 5.15 *R*^2^ = 0.8656	0.3149	2.86	15.55	42.84
308		0.4229	2.20		
318		0.4665	2.01		
**o-ECNFs**					
298	y = −3626.66x + 14.1 *R*^2^ = 0.9463	6.6500	−4.69	30.15	117.23
308		11.0000	−6.14		
318		14.2600	−7.03		
**melam-ECNFs**					
298	y = 11118.11x − 32.17 *R*^2^ = 0.8814	201.4300	−13.14	−92.43	−267.44
308		36.2200	−9.19		
318		19.4900	−7.85		
**P*m*PDA-ECNFs**					
298	y = −8146.18x + 28.35 *R*^2^ = 0.9969	2.8100	−2.56	67.73	235.72
308		6.4500	−4.77		
318		15.7200	−7.28		

Table 4 shows the comparison between different carbon materials as adsorbents for the removal of Pb^2+^ ions. According to the maximum adsorption capacity and the required time to equilibrium, functionalized ECNFs are a better nanoadsorbent for Pb^2+^ ions removal than most reported carbon nanomaterials.

**Table 4:** A comparison of the adsorption capacity of functionalized ECNFs with the other carbon nanomaterials.

Adsorbent	Adsorption capacity *q*_*max*_ (mg/g)	Condition	Ref.
CNTs	102	pH 5, time = 80 min	^51^
MWCNTs@SiO_2_-NH_2_	147	pH 7, *C*_*o*_ = 250 mg/L, T = 30 °C, time = 24 h	^52^
Oxidized MWCNTs	117.64	pH 7, *C*_*o*_ = 1200 mg/L, T = 25 °C	^53^
CNF-CoFe_2_O_4_	42.9	pH 4, *C*_*o*_ = 100 mg/L, T = 25 °C, time = 180 min	^54^
GO aerogels	158.7	pH 5.5, *C*_*o*_ = 100 mg/L,	^55^
magnetic chitosan@GO	76.94	pH 5, T = 30 °C	^56^
GO/L-Trp	222	pH 4, *C*_*o*_ = 250 mg/L, T = 20 °C, time = 40 min	^57^
ECNFs	105	pH 5.5, *C*_*o*_ = 200 mg/L, T = 25 °C, time = 180 min	This work
oxidized ECNFs	243	pH 5.5, *C*_*o*_ = 200 mg/L, T = 25 °C, time = 60 min	This work
melam-ECNFs	367.5	pH 5.5, *C*_*o*_ = 200 mg/L, T = 25 °C, time = 60 min	This work
P*m*PDA-ECNFs	354.5	pH 5.5, *C*_*o*_ = 200 mg/L, T = 25 °C, time = 60 min	This work

## Conclusion

Electrospun carbon nanofibers (ECNFs) can be fabricated *via* electrospinning followed by carbonization process. ECNFs showed superior over other carbon nanomaterials where their properties can be controlled during carbonization process. Moreover, their high surface area-to-volume, small fiber diameters and porosity, make them an attractive for various applications. The efficiency of ECNFs can be improved via surface modification to increase its hydrophilicity and hence their efficiency in removing heavy metals such as Pb^2+^ ions. Routine analysis confirmed the successful surface modification with melamine and P*m*PDA. The adsorption of Pb^2+^ ions onto the as-prepared materials, o-ECNFs, P*m*PDA-ECNFs, and Melam-ECNFs, is pH dependent and was better than onto pristine ECNFs. The maximum adsorption capacities (*Q°*_*max*_) of the ECNFs, o-ECNFs, P*m*PDA-ECNFs, and melam-ECNFs were 105, 243, 354, and 367 mg/g, respectively. Experimental data for the isotherms and kinetics fitted well with Langmuir isotherm model and PSO model, respectively. According to thermodynamic studies, the adsorption of Pb^2+^ ions onto functionalized ECNFs was spontaneous and endothermic, except with melam-ECNFs was exothermic process. The adsorption mechanism could be attributed to the functional groups on the surface of functionalized ECNFs, which could bind with Pb^2+^ ions through complexation or electrostatic interaction, while the adsorption mechanism on the surface of pristine ECNFs could be ascribed to nonspecific binding. Overall, the surface modification of the ECNFs successfully enhanced the adsorption capacity and the adsorption rate of Pb^2+^ ions from aqueous solutions.

## Experimental Methods

### Materials and reagents

*N,N’*-dicyclohexylcarbodiimide (DCC), polyacrylonitrile (PAN), lead nitrate (Pb(NO_3_)_2_), *m*-phenylene diamine, and melamine were provided from Sigma–Aldrich. *N,N*-dimethyl formamide (DMF) and hydrochloric acid were purchased from Panreac AppliChem. Nitric acid and sodium hydroxide were purchased from Global Chemical Ltd. Dimethyl sulfoxide (DMSO) was purchased from bio-Basic INC. *N*-hydroxysuccinimide (NHS) and *N*-(3-(dimethylamino)propyl)-*N’*-ethylcarbodiimide hydrochloride (EDC) were purchased from TCI (Tokyo, Japan). All chemicals were used without purification.

### Synthesis of adsorbents

ECNFs were prepared using electrospinning followed by carbonization, as described by Thamer *et al*.^27^. In a typical way, PAN (1 g) was dissolved in DMF (9 mL) with stirring at room temperature for 12 h. Then, the mixture was transferred to a syringe and electrospinning take place under the following conditions; an applied voltage of 16 kV; tip-to-collector distance (TCD) was 15 cm, and the humidity was >30%. PAN nanofibers were deposited on polyethylene paper on a drum collector and then the mat was removed and dried at 80 °C under vacuum for 12 h. PAN nanofibers were stabilized at 280 °C for 2 h under air followed by carbonization at 800 °C for 1.0 h under inert gas (nitrogen) with a heating rate of 5 °C/min.

**Scheme 1:**
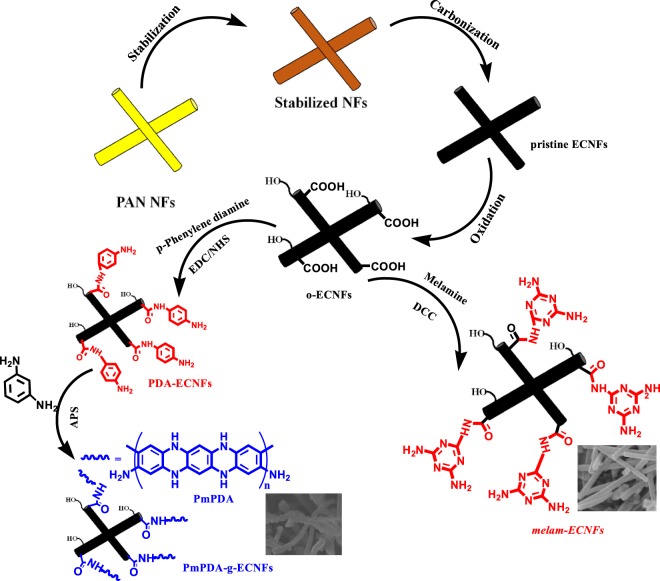
Fabrication steps of pristine and functionalized ECNFs.

Oxidized ECNFs (o-ECNFs) and poly(*m*-phenylene diamine)-*g*-ECNFs (P*m*PDA-ECNFs) were prepared according the procedure described elsewhere^29^ as shown in Scheme 1.

Melamine was grafted onto ECNFs *via* amidation reaction, as follows: o-ECNFs (250 mg) were dispersed in DMSO (50 mL) *via* sonication for 15 min. Then, the mixture was cooled in an ice bath, followed by addition of DCC (0.5 g) with stirring for 1 h. Melamine was added to the mixture, with stirring at room temperature for 48 h. The solid product (melam-ECNFs) was filtrated, washed several times with hot ethanol and dried at 70 °C under vacuum overnight as shown in Scheme 1.

### Characterization of adsorbents

The chemical structure and surface modification of the ECNFs was confirmed using diverse techniques, such as Fourier transform infrared (FT-IR) spectroscopy (Optic Tensor 27, Bruker, Germany), thermogravimetric analysis (TGA) (Q500, TA, USA), X-ray photoelectron spectroscopy (XPS, Al *K*_α_ X-ray,Thermo Scientific) and X-ray diffraction (XRD) measurements (MiniFlex) with Cu *K*_α_ radiation (λ = 1.5418 Å). The morphology of all the adsorbents was analyzed via field-emission scanning electron microscopy (FE-SEM, JEOL2100F). The nature of the surface charge was determined using a NanoPlus zeta/nanoparticle analyzer.

### Adsorption experiments

The initial Pb^2+^ ions concentrations varied from 25 to 200 mg/L, prepared from a stock solution of 1000 mg/L, and the pH value of all the Pb^2+^ ions solutions was maintained at 5.5.

For adsorption isotherm analysis, the adsorption study was conducted by adding 3 mg of adsorbent to 12 mL of lead ion (Pb^2+^) solution (in a polypropylene tube) at 25, 35, and 45 °C. After the adsorbent was added, the tube containing the solution was shaken for 4 h. The samples were filtered through a 0.4-μm membrane filter, and the residual concentrations of Pb^2+^ ions in the filtrates were determined using atomic absorption spectrometer (PerkinElmer, USA) at a wavelength of 283.3 nm. The effect of the pH on the Pb^2+^ ions removal by the adsorbents was investigated at pH values ranging from 3 to 7, which can be adjusted by adding NaOH or HNO_3_ and measured using a pH meter (3510-Jenway). Mixtures of 3 mg of adsorbent and 12 mL of Pb^2+^ ions (100 mg/L) with different pH values were shaken at 70 rpm and 25 °C for 24 h.

To determine the equilibrium contact time, a 200-mg/L solution of Pb^2+^ ions was exposed to the adsorbents as previously mentioned, and samples were removed at various times (from 10 to 720 min). Then, the mixture was filtered through a 0.4-μm-pore size membrane. The amounts of adsorbed Pb^2+^ ions at equilibrium (*q*_*e*_, mg/L) at time *t* (q_t_, mg/L) were calculated using the following equations:

7 $${q}_{e}=\frac{{C}_{o}-{C}_{e}}{m}\times V$$

8 $${q}_{t}=\frac{{C}_{o}-{C}_{t}}{m}\times V$$

where *C*_*o*_ (mg/L) represents the initial concentration of Pb^2+^ ions, *C*_*e*_ (mg/L) represents the equilibrium concentration in the solution, *V* (L) represents the volume of the solution, and *m* (g) represents the adsorbent mass.

## Supplementary information

Supplementary information
