# Supplementary material for: Fabrication of functionalized electrospun carbon nanofibers for enhancing lead-ion adsorption from aqueous solutions
Source: Sci Rep. 2019 Dec 19;9:19467. doi: 10.1038/s41598-019-55679-6 (PMC6923440; doi:10.1038/s41598-019-55679-6)
Supplement: Supplementary file 1 — Supplementary information [file 41598_2019_55679_MOESM1_ESM.docx]

**Fabrication of functionalized electrospun carbon nanofibers for enhancing lead-ion adsorption from aqueous solutions**

Badr M. Thamer^a^, Ali Aldalbahi^a^, Meera Moydeen A^a^, Abdullah M. Al-Enizi^a^, Hany El-Hamshary^a, b^ & Mohamed H. El-Newehy^a, b*^

*^a^ Department of Chemistry, College of Science, King Saud University, Riyadh 11451, Saudi Arabia*

*^b^ Department of Chemistry, Faculty of Science, Tanta University, Tanta 31527, Egypt*

*Corresponding author; e-mail address: melnewehy@science.tanta.edu.eg (M.H. El-Newehy)


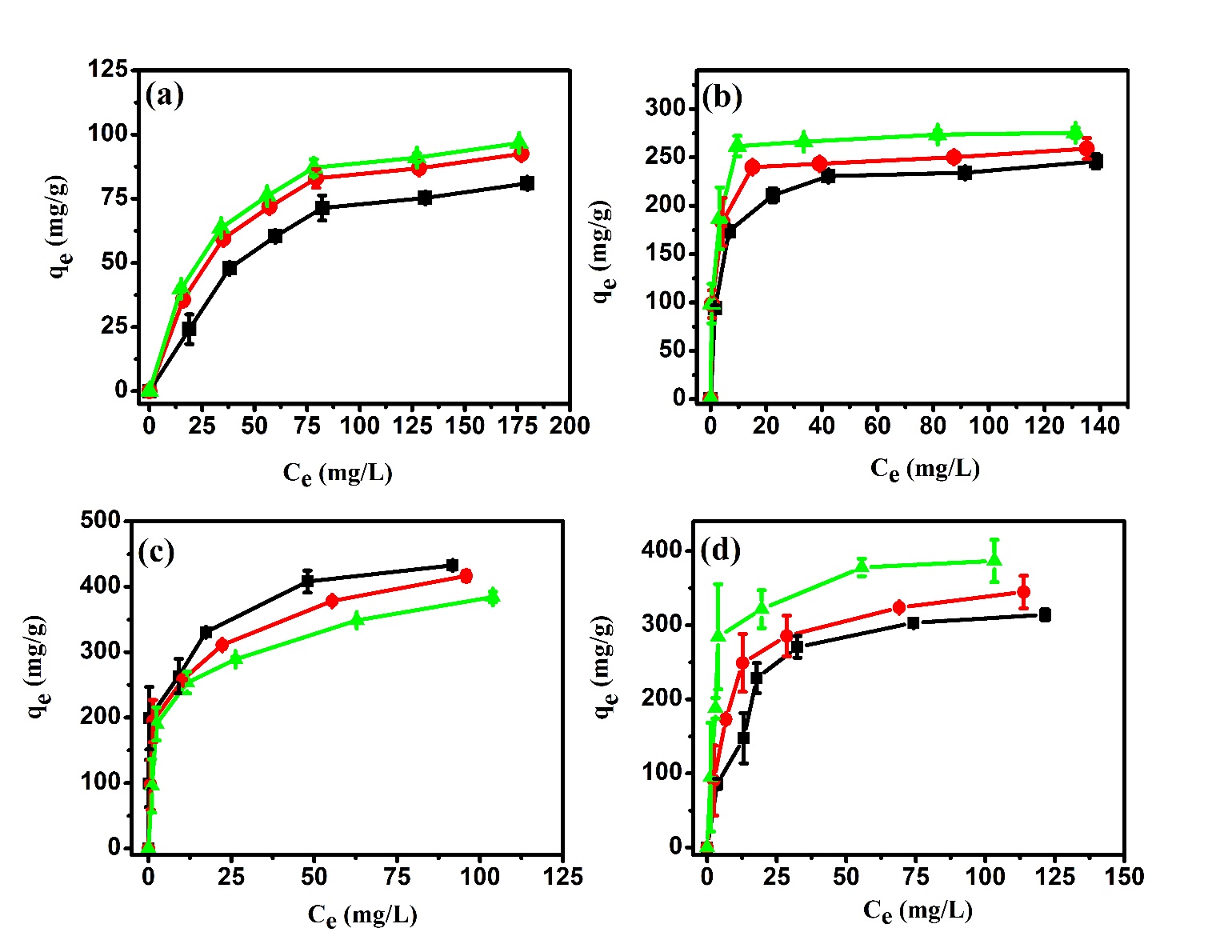


**Figure S1.** Isotherms for the adsorption of Pb^2+^ ions with 5 % error bars onto (a) ECNFs, (b) o-ECNFs, (c) Melam-ECNFs, and (d) P*m*PDA-ECNFs.


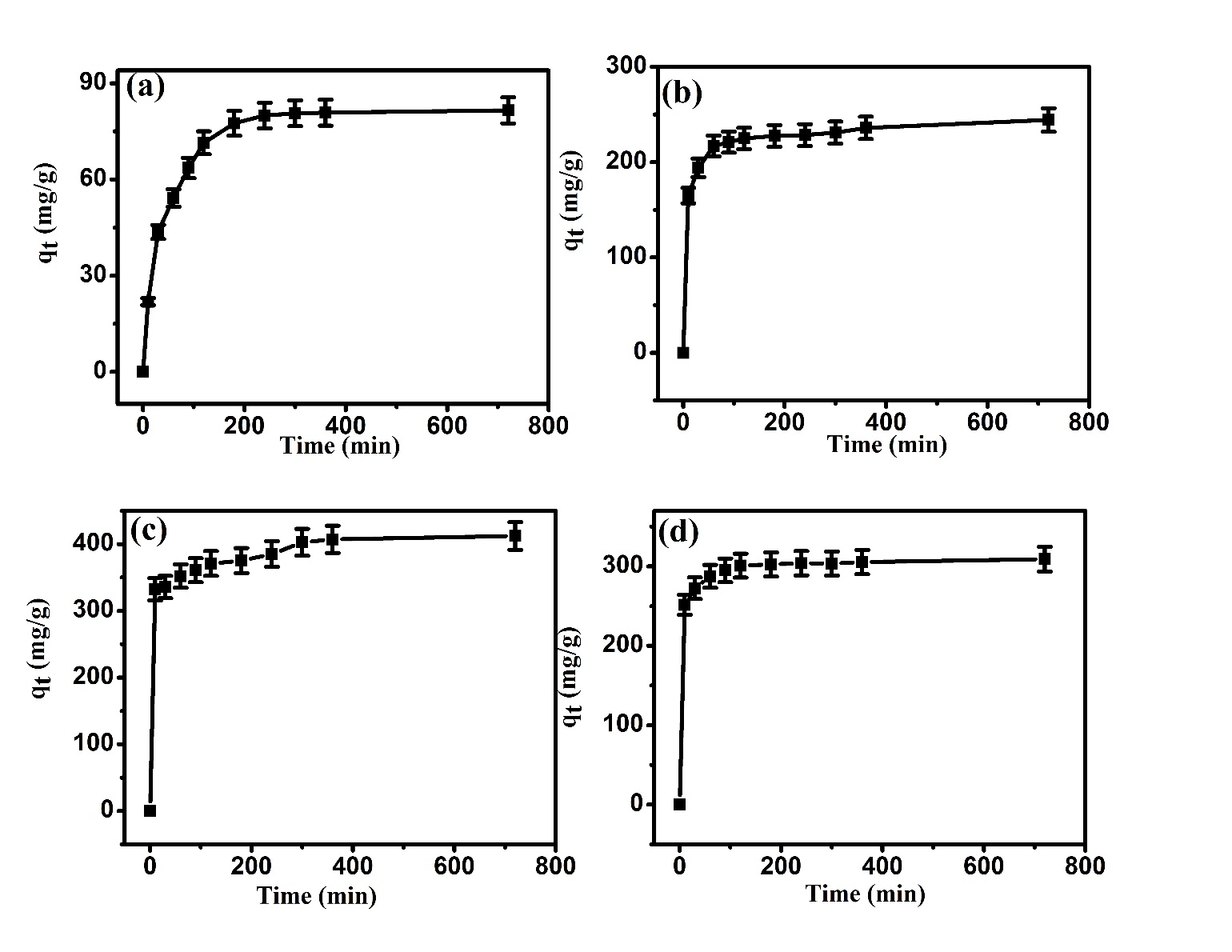


**Figure S2:** kinetic study of the adsorption of Pb^2+^ ions with 5 % error bars onto (a) ECNFs, (b) o-ECNFs, (c) melam-ECNFs, and (d) PmPDA-ECNFs.

**Table S1:** Surface area and pore structure of adsorbents.

| **Sample code** | **Surface area**  **(m^2^ g^-1^)** | **Pore volume**  **(cm^3^ g^-1^)** |
| --- | --- | --- |
| ECNFs | 10.1 | 0.022 |
| O-ECNFs | 115.6 | 0.029 |
| PmPDA-ECNFs | 7.7 | 0.081 |

**Section I: nonlinear isotherm models**

Langmuir model:

$$q_{e}=\frac{Q_{o}K_{L}C_{e}}{1+K_{L}C_{e}} (1)$$

Freundlich model:

$$q_{e}=K_{f} C_{e}^{n} (2)$$

Dubinin–Radushkevich model

$$q_{e}= Q_{o} e^{-K_{D-R} \varepsilon^{2}} (3)$$

$$\varepsilon=RTln(1+\frac{1}{C_{e}} ) (4)$$

$$E=\frac{1}{\sqrt{{2K}_{D-R}}} (5)$$

Here, *q_e_*, *C_e_*, *Q_o_*, and *K_L_* represent the amount of metal ions adsorbed onto the surface of the adsorbent (mg/g), the remaining concentration of metal ions at equilibrium (mg/L), the maximum adsorption capacity that covers the monolayer on the surface of the adsorbent, and the Langmuir constant (L/mg), respectively. *K_F_* [(mg/g)/(L/mg)*n*] is the Freundlich constant, which characterizes the strength of adsorption; *n* (dimensionless) is a Freundlich intensity parameter; *q_DR_* (mg/g) represents the adsorption capacity; *K_DR_* (mol^2^/kJ^2^) is a constant related to the sorption energy; *ɛ* represents the Polanyi potential; and *E* (kJ/mol) represents the mean adsorption energy.

**Section II: nonlinear Kinetic models**

Pseudo-first-order model:

$$q_{t}=q_{e}\left( 1-e^{{-K}_{1}t} \right) (6)$$

Pseudo-second-order model:

$$q_{t}=\frac{q_{2}^{2}k_{2}t}{1+ q_{e}K_{2}t} (7)$$

Elovich model:

$$q_{t}=\frac{1}{\beta}\ln\left( 1+\alpha\beta t \right) (8)$$

Here, *ԛ_t_* and *q_e_* represent the amounts of Pb^2+^ adsorbed at time *t* and at equilibrium, respectively; *K_1_* (min^-1^) and *K_2_* (g/mg min) are the rate constants of the PFO and PSO adsorption process, respectively; *α* (mg/g min) represents the initial adsorption rate; and *β* (mg/g) is the desorption constant during an experiment.

**Section III: thermodynamic parameters**

The free energy (∆*G*°) was calculated using Eq. (11), enthalpy (∆*H*°) and entropy (∆*S*°) were calculated using the slope and intercept, respectively, of the linear plot of ln*K_c_* vs. 1/*T*.

$\Delta G^{\circ}=-RTlnKc$ (9)

$lnK_{c}=\frac{\Delta S^{\circ}}{R}-\frac{\Delta H^{\circ}}{RT}$ (10)

Here, *R* and *T* represent the universal gas constant (8.3144 J/mol K) and the absolute temperature (K), respectively. *K_c_* is the thermodynamic equilibrium constant, i.e., the ratio of the amount of dye on the adsorbent at equilibrium (*C_ad_*) to the residual dye concentration in the solution at equilibrium (*C_e_*).
